# DNA methylation markers for cancer risk prediction of vulvar intraepithelial neoplasia

**DOI:** 10.1002/ijc.33459

**Published:** 2021-01-19

**Authors:** Nikki B. Thuijs, Johannes Berkhof, Müjde Özer, Sylvia Duin, Annina P. van Splunter, Barbara C. Snoek, Daniëlle A. M. Heideman, Marc van Beurden, Renske D. M. Steenbergen, Maaike C. G. Bleeker

**Affiliations:** ^1^ Pathology, Cancer Center Amsterdam Amsterdam UMC, Vrije Universiteit Amsterdam Amsterdam The Netherlands; ^2^ Epidemiology and Data Science Amsterdam UMC, Vrije Universiteit Amsterdam Amsterdam The Netherlands; ^3^ Plastic, Reconstructive and Hand Surgery Amsterdam UMC, Vrije Universiteit Amsterdam Amsterdam The Netherlands; ^4^ Department of Gynecology Antoni van Leeuwenhoek hospital Amsterdam The Netherlands

**Keywords:** biomarker, DNA methylation, human papillomavirus, vulvar intraepithelial neoplasia, vulvar squamous cell carcinoma

## Abstract

Current clinical and histological classifications are unable to determine the risk of vulvar squamous cell carcinoma (VSCC) in high‐grade vulvar intraepithelial neoplasia (VIN), making prognostic biomarkers highly needed. We studied host‐cell DNA methylation markers in high‐grade squamous intraepithelial lesion (HSIL) and differentiated VIN (dVIN) without VSCC, in HSIL and dVIN adjacent to VSCC and in human papillomavirus (HPV) positive and negative VSCC, relative to control vulvar tissues. A series of 192 formalin‐fixed paraffin‐embedded vulvar samples, including VSCC (n = 58), VIN adjacent to VSCC (n = 30), VIN without VSCC during follow‐up (n = 41) and normal vulvar tissues (n = 63), were tested for 12 DNA methylation markers with quantitative multiplex methylation‐specific PCR (qMSP). HPV status was determined by p16^INK4A^ immunohistochemistry and high‐risk HPV PCR analysis. Logistic regression analyses were used to determine methylation patterns and methylation marker performance for VIN and VSCC detection. Methylation markers showed significantly higher methylation levels with increasing severity of disease. VIN adjacent to VSCC showed a similar methylation‐high pattern as VSCC, while VIN without VSCC displayed a heterogeneous methylation pattern. Vulvar carcinogenesis is associated with increased DNA methylation. Higher DNA methylation levels in VIN seem to reflect higher cancer risk, emphasizing the high potential of DNA methylation biomarkers in the diagnostic workup of VIN. As a next step, longitudinal studies are needed to verify the prognostic value of methylation biomarkers as a clinical tool for stratification of cancer risk in women with VIN.

## Abstract 2

**What's new?**

In high‐grade vulvar intra‐epithelial neoplasia (VIN), existing clinicopathological classifications lack accuracy in predicting cancer risk, resulting in a need for objective prognostic biomarkers. Here, 12 DNA methylation markers were investigated for prognostic capability in a series of high‐grade squamous intraepithelial lesion, differentiated VIN, and vulvar carcinomas. For all 12 methylation markers, as disease severity increased, methylation levels also increased. Methylation patterns were more heterogeneous for VIN without vulvar squamous cell carcinoma. The results link elevated DNA methylation levels with increased VIN cancer risk and indicate that methylation biomarkers are promising diagnostic tools for cancer risk stratification in VIN.

**Abbreviations**
ACTB: β‐actin
AIN: anal intraepithelial neoplasia
AUC: area under the curve
CIN: cervical intraepithelial neoplasia
Ct: cycle threshold
dVIN: differentiated VIN
FFPE: formalin‐fixed, paraffin embedded
FIGO: International Federation of Gynecology and Obstetrics
HPV: human papillomavirus
HSIL: high‐grade squamous intraepithelial lesion
qMSP: quantitative multiplex methylation‐specific PCR
ROC: receiver operating characteristic
UVIN: usual type of vulvar intraepithelial neoplasia
VIN: vulvar intraepithelial neoplasia
VSCC: vulvar squamous cell carcinoma

## 1 INTRODUCTION

Vulvar squamous cell carcinoma (VSCC) accounts for approximately 5% of gynecological malignancies and 95% of all vulvar malignancies. The precursor lesion of VSCC is high‐grade vulvar intraepithelial neoplasia (VIN). VIN is classified into high‐grade squamous intraepithelial lesion (HSIL), which is human papillomavirus (HPV) related, and differentiated VIN (dVIN), which is independent of HPV and associated with lichen sclerosus.^1^, ^2^, ^3^ HSIL, previously known as usual type of VIN (uVIN), is the most common type of VIN, occurring mainly in women aged between 35 and 50 years. Treatment modalities range from topical imiquimod to surgery, leading to somatic and psychosexual morbidity.^4^ Despite the relatively low absolute cancer risk of HSIL, that is, 2.3% to 6.6% after 3 years, all HSIL are treated to prevent cancer.^5^, ^6^, ^7^ Current clinicopathological parameters are insufficient to accurately predict individual cancer risk. To reduce overtreatment and associated morbidity, biomarkers that could predict individual cancer risk in women with HSIL are urgently needed.

The molecular events leading to the development of VSCC through VIN are not yet well understood. Few studies have examined DNA mutation or copy number alterations and correlated these with the risk of progression in VIN, but no prognostic biomarkers ready for clinical use have been found so far.^8^, ^9^ Epigenetic changes, such as hypermethylation of promoter cytosine‐phosphate‐guanine islands of tumor suppressor genes, can contribute to the development of cancer by gene silencing.^10^ In HPV‐related cervical and anal disease, DNA methylation testing has provided promising biomarkers for the identification of precursors with a presumed high cancer risk.^10^, ^11^, ^12^, ^13^ Various methylation markers associated with HPV‐induced anogenital carcinogenesis have been discovered, including *ASCL1*, *CADM1*, *FAM19A4*, *GHSR*, *LHX8*, *MAL*, *miR124‐2*, *PHACTR3*, *PRDM14*, *SST*, *ZIC1* and *ZNF582*.^12^, ^14^, ^15^ In vulvar (pre)malignancies, few data exist on DNA methylation of host cell genes.

In our study, we tested above 12 methylation markers in a large and well‐defined series of HPV positive and negative vulvar carcinomas and VIN, divided into VIN without progression to VSCC during long‐term follow‐up and VIN adjacent to VSCC, to assess the potential value for cancer risk prediction of VIN.

## 2 MATERIALS AND METHODS

### 2.1 Patients and samples

Our study included 192 vulvar samples from 192 women, categorized into 4 groups: normal (control) vulvar tissues (n = 63), VIN without VSCC (n = 41), VIN adjacent to VSCC (n = 30) and VSCC (n = 58). VIN without VSCC refers to VIN lesions detected in women that did not develop VSCC during a median follow‐up time of 17.8 years (range, 1.0‐27.1 years). To confirm the absence of VSCC, follow‐up data with nationwide coverage were retrieved from the nationwide network and registry of histopathology and cytopathology in the Netherlands.^16^ The group of VIN adjacent to VSCC was used as surrogate for the most advanced stage of VIN, representing VIN with a high progression risk to cancer. HSIL, dVIN and VSCC tissues were retrieved from the pathology archives of Amsterdam UMC and Antoni van Leeuwenhoek hospital, in Amsterdam, the Netherlands, between 1984 and 2015. Compared to regular care, VIN adjacent to VSCC and VSCC were enriched for HPV‐positive cases.^9^ The control group comprised vulvar samples from healthy patients collected during esthetic genital procedures in the “V Klinieken” in Leiden, the Netherlands, or during reconstructive genital procedures in Amsterdam UMC, location VUmc, in 2018 and 2019.

### 2.2 Histopathology

Formalin‐fixed, paraffin embedded (FFPE) tissue blocks were sectioned using the sandwich method. The first and last sections (3 μm) were used for hematoxylin‐eosin staining to ensure the presence of the same lesion, and in‐between sections (10 μm) were collected in sterile PCR tubes for DNA isolation. Precautions were taken to avoid cross‐contamination as described before.^17^

VIN adjacent to VSCC samples were selected in women with VSCC with sufficient adjacent VIN. VIN adjacent to VSCC and VSCC were harvested by laser‐capture microdissection when present in one tissue block. For the selection of tissues, all slides were reviewed by a gynecopathologist (Maaike C. G. Bleeker) and a senior resident in pathology (Nikki B. Thuijs). Histological subtypes of VIN (HSIL or dVIN) and VSCC (keratinizing or basaloid/warty) as well as the International Federation of Gynecology and Obstetrics (FIGO) stage of all VSCC cases were documented.

### 2.3 DNA isolation

DNA was isolated using the QIAamp DNA FFPE tissue kit (Qiagen, Hilden, Germany) according to the manufacturer's instructions and was eluted with the easyMAG 3 elution buffer (bioMérieux, Boxtel, the Netherlands). DNA concentration was measured using Qubit (Thermo Fisher Scientific Inc, Qiagen).

### 2.4 DNA methylation analysis using quantitative multiplex methylation‐specific PCR (qMSP)

DNA was bisulfite‐converted using the EZ‐DNA Methylation kit (Zymo Research, Orange, CA).^18^ For methylation analysis, EpiTect MethyLight Master Mix (Qiagen) was used, together with fluorescent dye‐labeled probes, 50 ng of bisulfite‐converted DNA and 100‐300 nM of each primer.^19^

We analyzed 12 DNA methylation markers in four multiplex qMSP assays, each assay targeting three markers and the reference gene, β‐actin (*ACTB*).

Multiplex qMSPs targeting *GHSR/SST/ZIC1* and *ASCL1/LHX8/ZNF582* were performed on the ViiA7 Real‐Time PCR System with inclusion of a calibrator (Applied Biosystems, Foster City, CA) and multiplex qMSPs targeting *FAM19A4/PHACTR3/PRDM14* and *CADM1/MAL/miR124‐2* were run on the ABI 7500 Fast Real‐Time PCR System (Applied Biosystems).^18^, ^19^, ^20^ All samples were first tested for the six markers *ASCL1*, *LHX8*, *ZNF582*, *GHSR*, *SST* and *ZIC1*. Due to limited availability of DNA, multiplex qMSP *CADM1/MAL/miR124‐2* was tested on 129/192 samples and multiplex qMSP *FAM19A4/PHACTR3/PRDM14* on 143/192 samples. A Ct ≤32 for *ACTB* indicated sufficient DNA and adequate bisulfite conversion.^20^ Invalid test results (ie, *ACTB* Ct >32) were obtained from 3/129 samples tested for qMSP *CADM1/MAL/miR124‐2*. No invalid results were obtained from the remaining three multiplexes.

ΔCt or ΔΔCt ratios were computed using the comparative Ct method, normalizing target Ct values to respectively *ACTB* or to *ACTB* and a calibrator.^21^

### 2.5 HPV status

Immunostaining of p16^INK4a^ was performed with mouse monoclonal antibodies against the p16^INK4a^ antigen (clone E6H4; Roche, Basel, Switzerland), using the Optiview detection kit with the automated BenchMark ULTRA IHC/ISH system (Roche). p16^INK4A^ immunohistochemistry was scored positive when diffuse or block staining was observed and negative with a negative or patchy staining pattern.^22^

High‐risk HPV DNA‐testing was performed using the QIAscreen HPV PCR Test (Qiagen), as described previously for use on FFPE biopsy specimens.^23^ The assay is directed against the E7 gene of 15 (probably) high‐risk HPV genotypes, that is, 16, 18, 31, 33, 35, 39, 45, 51, 52, 56, 58, 59, 66, 67 and 68, with partial genotype information (HPV16 and −18).^24^ Beta‐globin served as internal quality control. Samples were considered invalid for PCR testing when the cycle threshold (Ct) >30 for beta‐globin and no HPV was found.

HPV status was determined in all VIN and VSCC and not in controls. HPV status was considered positive when p16^INK4A^ and/or HPV PCR were positive, and negative when p16^INK4A^ was negative and HPV PCR was negative or invalid.

### 2.6 Statistical analysis

To evaluate methylation levels per disease category, boxplots were computed from the log2‐transformed Δ(Δ)Ct ratios of the markers. Differences in methylation levels between disease categories were assessed using the Kruskal‐Wallis test, followed by post hoc testing using the Mann‐Whitney *U* test and by Bonferroni multiple testing correction in cases with significant results.

Univariable logistic regression analyses were performed on log2‐transformed Δ(Δ)Ct ratios of 6/12 markers with complete methylation data (*ASCL1*, *LHX8*, *ZNF582*, *GHSR*, *SST* and *ZIC1*). A logistic regression model built for normal vs VSCC was used to visualize methylation patterns by calculating predicted probabilities of underlying VSCC for each sample and marker, with values ranging from 0 to 1. To assess the potential diagnostic value of the six methylation markers for the clinical management of women with VIN, we compared VIN without VSCC vs controls and VIN without VSCC vs VSCC by visualizing receiver operating characteristic (ROC) curves, assessed through the area under the curve (AUC).

Logistic regression analysis was performed in R open source software version 4.0.2 and the pROC package was implemented for ROC analysis. All other statistical analyses were performed in IBM SPSS Statistics software for Windows version 24.0 (IBM Corporation, Armonk, NY). Reported p values were 2‐sided. *P* < .05 was considered statistically significant and was scored as marginal evidence (.01 < *P <* .05), moderate evidence (.001 < *P* < .01) and strong evidence (*P* < .001).

## 3 RESULTS

### 3.1 Baseline characteristics

Baseline characteristics and HPV status per disease category of the study population are shown in Table 1. Median age was highest for patients with VSCC (72.5 years; range, 36‐95) and lowest for controls (28.0 years; range, 18‐57). FIGO stages of the VSCCs were stage Ia in 4, Ib in 33, IIIa in 10, IIIb in 2 and IIIc in 9 tumors.

**TABLE 1:** Baseline characteristics and HPV status per disease category

		Control	VIN		VSCC
			Without VSCC	Adjacent to VSCC	
Number		63	41	30	58
Median age (range)		28.0 (18‐57)	42.0 (21‐86)	66.0 (36‐92)	72.5 (36‐95)
Histological subtype of VIN (%)	HSIL		37 (90.2)	18 (60.0)	
	dVIN		4 (9.8)	12 (40.0)	
Histological subtype of VSCC (%)	Keratinizing				41 (70.7)
	Basaloid/warty				17 (29.3)
HPV status (%)	Positive		37 (90.2)	18 (60.0)	27 (46.6)
	HPV16		29 (70.7)	12 (46.7)	26 (44.8)
	HPV18		0 (0)	0 (0)	0 (0)
	Non‐16/18 high‐risk HPV type		7 (17.1)	4 (20.0)	0 (0)
	HPV16 and non‐16/18 high‐risk HPV type		1 (2.4)	2 (6.7)	0 (0)
	Not determined		0 (0)	0 (0)	1 (1.7)
	Negative		4 (9.8)	12 (40.0)	31 (53.4)

Abbreviations: dVIN, differentiated VIN; HPV, human papillomavirus; HSIL, high‐risk squamous intraepithelial lesion; VIN, high‐grade vulvar intraepithelial neoplasia; VSCC, vulvar squamous cell carcinoma.

HPV status was positive in 90.2% (37/41) of VIN without VSCC, in 60.0% (18/30) of VIN adjacent to VSCC and in 46.6% (27/58) of VSCC. All HPV‐positive VIN had HSIL morphology and all HPV‐negative VIN had dVIN morphology. The keratinizing and the basaloid/warty subtype of VSCC was found in 59.3% and 40.7% of the HPV‐positive VSCCs and in 83.9% and 16.1% of the HPV‐negative VSCCs, respectively. Predominant HPV genotype was HPV16, accounting for respectively 80.6% and 100% of all HPV‐positive VIN without VSCC and VSCC. Multiple infections were found in 3.8% (3/80) of HPV PCR‐positive samples.

### 3.2 DNA methylation levels in different vulvar disease categories

**FIGURE 1:**
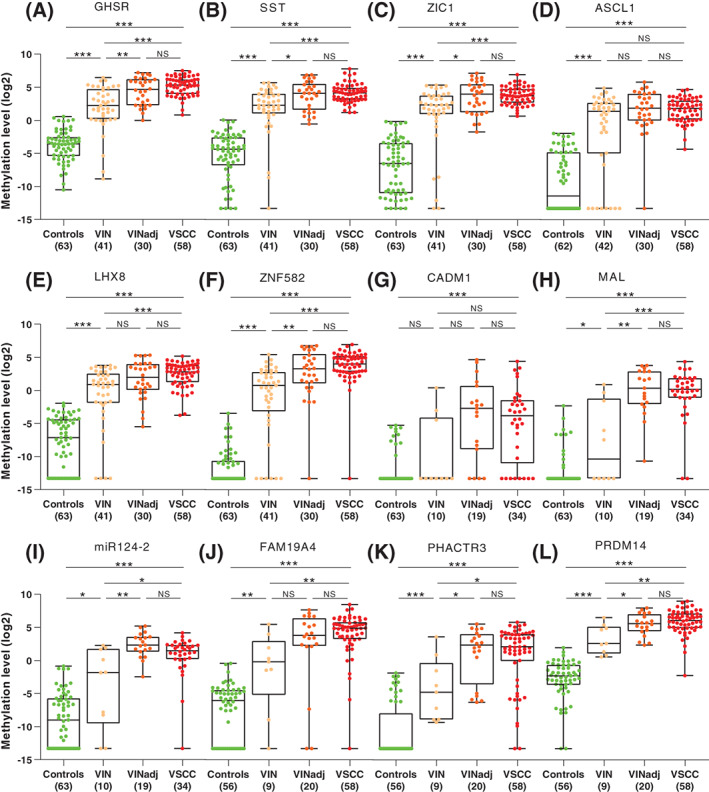
DNA methylation levels shown relative to the reference gene ACTB (log2‐transformed Δ(Δ)Ct ratios; y‐axis) for the 4 disease categories (x‐axis) for 12 markers: A, GHSR; B, SST; C, ZIC1; D, ASCL1; E, LHX8; F, ZNF582; G, CADM1; H, MAL; I, miR124‐2; J, FAM19A4; K, PHACTR3; and L, PRDM14. Differences between histological categories upon Kruskal‐Wallis test, followed by post hoc testing using the Mann‐Whitney *U* test and Bonferroni multiple testing correction: *P < .05 (marginal evidence), **P < .01 (moderate evidence), ***P < .001 (strong evidence), NS, not significant. VIN, high‐grade vulvar intraepithelial neoplasia; VINadj, VIN adjacent to VSCC; VSCC, vulvar squamous cell carcinoma [Color figure can be viewed at wileyonlinelibrary.com]

Methylation levels of 11/12 markers (except for CADM1) increased significantly with severity of disease. Significantly higher methylation levels were found for all markers in VSCC compared to controls, for 11/12 markers in VIN without VSCC compared to controls, for 10/12 markers in VIN without VSCC compared to VSCC, and for 8/12 markers in VIN without VSCC compared to VIN adjacent to VSCC (Figure 1). None of the markers showed a significant difference between VIN adjacent to VSCC and VSCC.

### 3.3 DNA methylation levels in relation to HPV status

In HPV‐positive samples, 10/12 markers (except for CADM1 and MAL) showed significantly higher methylation levels with increasing severity of disease (Supplementary Figure 1). For CADM1 and MAL, a trend toward higher methylation levels with increasing severity of disease was seen, but significance was not reached, likely because of small sample sizes.

In HPV‐negative samples, all markers showed significantly higher methylation levels with increasing severity of disease (Supplementary Figure 2). However, dVIN without VSCC was not tested for six markers (*CADM1*, *MAL*, *miR124‐2*, *FAM19A4*, *PHACTR3* and *PRDM14*), due to limited DNA availability.

### 3.4 Methylation patterns and diagnostic performance of individual methylation markers

**FIGURE 2:**
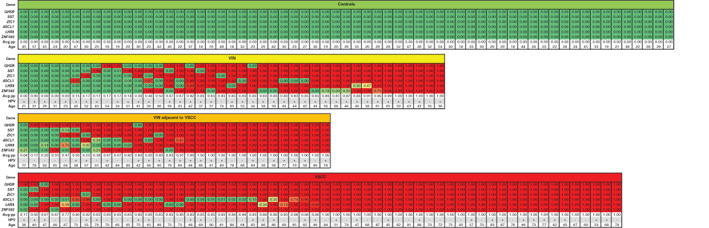
DNA methylation pattern of six methylation markers (GHSR, SST, ZIC1, ASCL1, LHX8 and ZNF582) across all four histological disease categories. Predicted probabilities (pp) per sample (column) are colored from green (pp of 0, ie, low) to red (pp of 1, ie, high). In each disease category, samples are ordered based on their average pp (Avg pp). HPV status of the samples and age of the patients is displayed at the bottom of each disease category. +, HPV positive; −, HPV negative; VIN, high‐grade vulvar intraepithelial neoplasia; VSCC, vulvar squamous cell carcinoma [Color figure can be viewed at wileyonlinelibrary.com]

The DNA methylation patterns, depicted by predicted probabilities of underlying VSCC for each sample separately, are shown in Figure 2. Controls uniformly showed very low predicted probabilities, consistent with a methylation‐low pattern. VSCCs showed uniformly high predicted probabilities, consistent with a methylation‐high pattern, with the lowest average predicted probability of the six markers equal to 0.17. Predicted probabilities were also consistently high across markers, with the exception of ASCL1, showing relatively low predicted probabilities in VSCC. VIN adjacent to VSCC showed predominantly high average predicted probabilities, similar to VSCC. VIN without VSCC demonstrated a heterogeneous methylation pattern, with samples displaying both low and high individual predicted probabilities (respectively green and red boxes in Figure 2).

Within individual disease categories, age and HPV were equally represented across low and high average predicted probabilities, with the exception of VIN adjacent to VSCC, in which the lowest five average predicted probabilities were found in dVIN.

**FIGURE 3:**
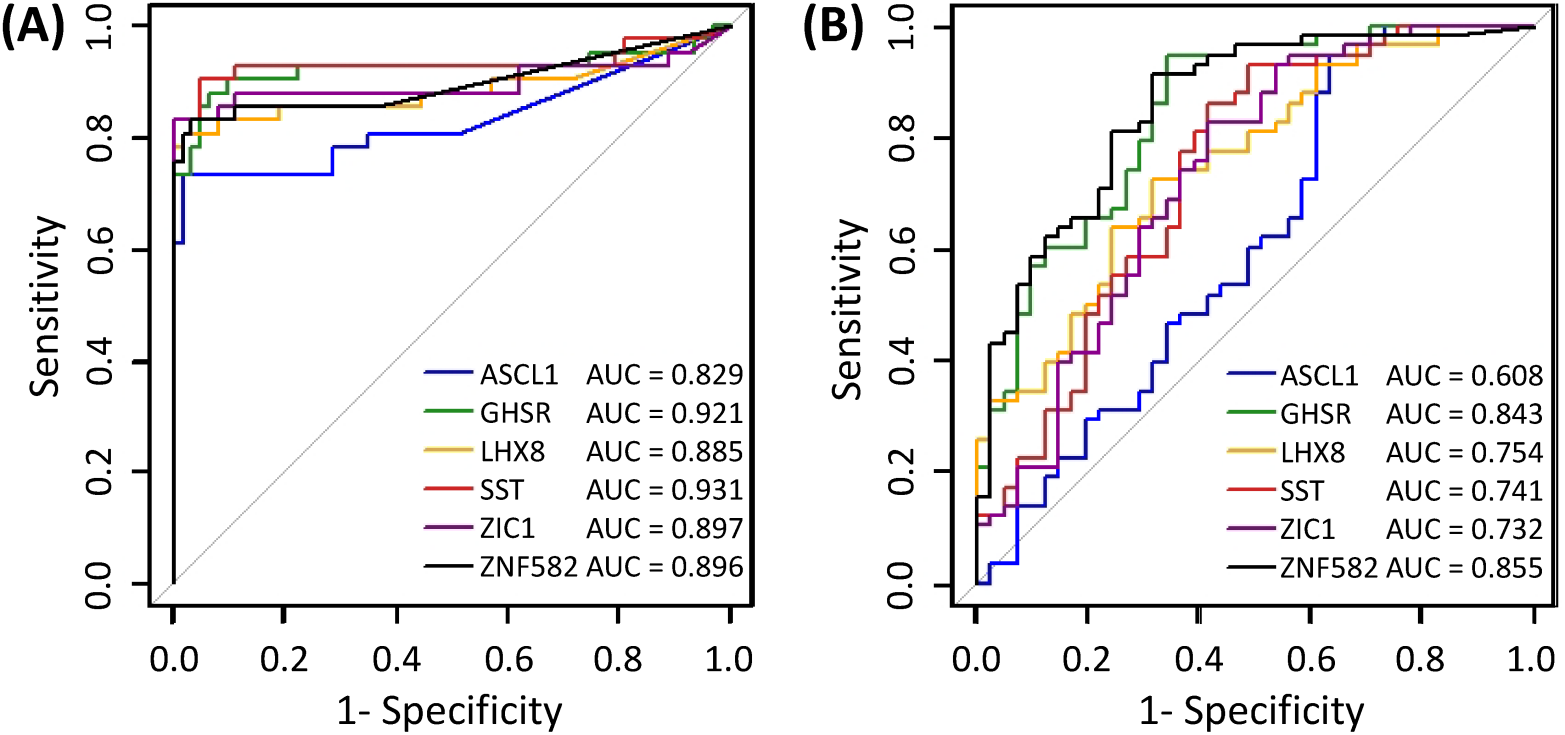
Diagnostic performance of six markers (GHSR, SST, ZIC1, ASCL1, LHX8 and ZNF582) for the ability to distinguish VIN without VSCC from controls (A) and VIN without VSCC from VSCC (B), assessed by univariable logistic regression analysis and visualized with ROC curves and AUCs. AUC, area under the curve; ROC, receiver operating characteristics; VIN, high‐grade vulvar intraepithelial neoplasia; VSCC, vulvar squamous cell carcinoma [Color figure can be viewed at wileyonlinelibrary.com]

Marker‐specific ROC curves demonstrated AUCs of 0.829 to 0.931 when discriminating between VIN without VSCC and controls (Figure 3A), and AUCs of 0.601 to 0.855 when discriminating between VIN without VSCC and VSCC (Figure 3B).

## 4 DISCUSSION

The most important outcome of our study is the significant increase in methylation levels with severity of disease and clearly distinct methylation patterns in VIN with different cancer risk. VIN adjacent to VSCC revealed equally high methylation levels as VSCC. Contrarily, VIN without VSCC displayed a heterogeneous methylation pattern characterized by either low or high methylation levels, suggestive of a variable cancer risk. Our results demonstrate that DNA methylation of the 12 genes studied is associated with vulvar carcinogenesis, with highly comparable results for both HPV‐induced and HPV‐independent oncogenic pathways. Altogether, these methylation markers may provide valuable biomarkers for risk stratification of VIN.

To our knowledge, our study examining 12 host‐cell DNA methylation markers in 192 vulvar samples, including 41 well‐defined VIN lesions without progression to VSCC during long‐term follow‐up and 30 VIN lesions adjacent to vulvar carcinoma, is the largest of its kind and the first to present results on these methylation markers in vulvar lesions. A correlation between increased methylation of specific markers and increasing severity of vulvar disease has already been described for a few other markers.^25^, ^26^, ^27^, ^28^, ^29^, ^30^, ^31^, ^32^, ^33^, ^34^, ^35^, ^36^ Only the markers *MGMT* and *p16* ^*INK4a*^ have been investigated more than once. Methylation of *p16* ^*INK4a*^ was commonly detected in both VIN and VSCC in six out of seven studies, while one study showed absence of *p16* ^*INK4a*^ methylation in all five vulvar carcinomas studied.^26^, ^27^, ^29^, ^30^, ^32^, ^33^, ^35^ *MGMT* methylation has been detected in 45% (13/20) and 36.7% (11/30) of vulvar carcinomas.^33^, ^37^ In comparison, in our series 98.3% (57/58) of carcinomas showed a methylation‐high pattern.

We have demonstrated that VIN adjacent to VSCC, considered as end stage VIN, displayed similarly high methylation levels as VSCC. It can be hypothesized that in VIN without VSCC high DNA methylation levels reflect a high cancer progression risk. The methylation‐high patterns seen in a subset of VIN without VSCC, can be explained by the fact that VIN is usually not diagnosed until a late stage, when symptoms have already developed. Adequate treatment of such lesions may have prevented cancer development. The observed varying methylation patterns in VIN without VSCC are consistent with the molecular heterogeneity described for copy number alterations and gene expression profiles in VIN.^9^ This molecular heterogeneity might in part explain why only a subset of VIN progress to cancer. Ideally, methylation biomarkers could guide clinical management with a more aggressive treatment for patients with VIN with many (epi)genetic alterations or methylation‐high patterns, while more conservative strategies can be chosen for patients with VIN with low methylation levels. Clinical guidance by additional use of methylation biomarkers could therefore potentially decrease harms of treatment and associated psychosexual sequelae.^4^

Heterogeneous methylation patterns of the genes studied have also been described in other studies on anogenital disease.^10^, ^13^, ^38^ In cervical scrapings of patients with cervical intraepithelial neoplasia grade 3 (CIN3) methylation levels were found to be linked to duration of disease existence, as was based on duration of the preceding high‐risk HPV infection. More advanced CIN3 lesions, with a presumed high cancer progression risk, showed high methylation levels, equal to cervical cancers. On the other hand, the so‐called early CIN3 lesions with a lower risk of progression to cancer were generally characterized by low methylation levels.^11^, ^12^, ^38^, ^39^ Similar findings have been described in high‐grade anal intraepithelial neoplasia (AIN) of HIV‐positive men having sex with men, also revealing heterogeneous methylation patterns with a subset of high‐grade AIN resembling anal cancer.^13^, ^19^ In contrast to the methylation patterns seen in CIN or AIN, characterized by a gradual range of average predicted probabilities, predicted probabilities in VIN without VSCC were either low or high.^19^, ^38^ The predicted probability model using VSCC samples as cases and healthy vulvar tissues as controls explains the dichotomy observed in our series.

One VSCC sample showed low individual predicted probabilities for five of six markers. This sample was HPV16 positive and was diagnosed in a 36‐year‐old woman, which is a remarkably low age for vulvar cancer. Studies have described an age‐associated increase in methylation levels.^40^, ^41^ However, in our study we found increased methylation levels in both young and older patients and therefore solely age is unlikely to explain the low methylation pattern in this case.

All our markers showed a very good performance, indicated by high AUCs, for the distinction between VIN without VSCC and controls (AUC 0.829‐0.931), and between VIN without VSCC and VSCC (AUC 0.608‐0.855). These results may be biased by our sample selection and the composition of the disease categories, because disease category sizes were not corrected for actual disease prevalence. Accordingly, no conclusions regarding clinical performance or optimal marker combinations can be drawn yet.

Our study has multiple strengths. This is the largest study in terms of markers and sample size, covering the complete spectrum of vulvar neoplasia. Controls were collected from healthy women resulting in uniform low methylation levels. VIN adjacent to VSCC was used as surrogate for VIN with high cancer risk, which we believe is a first necessary step in the exploration of methylation biomarkers for risk stratification of VIN. Our results on VIN adjacent to VSCC demonstrate that high methylation levels are likely linked to VSCC development. Also, we demonstrated a good performance of our markers in both HPV‐positive and ‐negative samples, in line with some of the markers also being methylated in other non–HPV‐induced cancers.^42^, ^43^

Our study has several limitations. Since we analyzed VIN adjacent to VSCC instead of VIN lesions showing progression to VSCC during follow‐up, we cannot prove VIN with a methylation‐high pattern do, indeed, have a higher risk of progression to cancer than their counterparts with a methylation‐low pattern. Second, the majority of VIN without VSCC (ie, 37/41) were HSIL, while only 4/41 were dVIN. The low number of dVIN in this group is explained by the fact that most dVINs are recognized at time of VSCC diagnosis and not prior to VSCC diagnosis. Third, due to DNA limitations not all markers could be tested on all samples. Nevertheless, a similar trend in methylation levels per disease category was observed for all 12 markers. Fourth, across disease categories, median age of the patients differed, which might have influenced the methylation levels. However, the age in our series reflects age distribution seen in regular care.^5^ Moreover, the effect of age on methylation levels is probably much weaker than the effect of strong biological processes involved in vulvar carcinogenesis.^41^

In conclusion, our study examining 12 DNA methylation markers revealed that methylation levels significantly increased from healthy vulvar tissue toward vulvar cancer. Histopathologically similar VIN without VSCC lesions displayed a heterogeneous methylation pattern. The methylation‐high pattern found in a subset of VIN and VIN adjacent to VSCC indicates the promising value of host‐cell DNA methylation testing to distinguish between VIN with low or high cancer progression risk. This is especially true for women with HSIL, in whom cancer risk stratification is clinically relevant. Future studies should include patients with VIN with variable clinical outcomes and long‐term follow‐up data to further evaluate the potential value of these methylation biomarkers for cancer risk stratification.

## CONFLICT OF INTEREST

Daniëlle A. M. Heideman and Renske D. M. Steenbergen are minority shareholders of Self‐screen B.V., a spin‐off company of VUmc. Self‐screen B.V. holds patents related to this work, and develops, manufactures and licenses the high‐risk HPV assay and methylation marker assays for cervical cancer. Daniëlle A. M. Heideman has been on the speakers' bureau of Qiagen and serves occasionally on the scientific advisory boards of Pfizer and Bristol‐Myers Squibb. All the other authors declared no potential conflicts of interest.

## ETHICS STATEMENT

The local Medical Ethics Committee of Amsterdam UMC, location VUmc, confirmed that the Medical Research Involving Human Subjects Act did not apply to this study and approved the study under reference numbers 2017.561 (VIN and VSCC) and 2017.626 (controls).

## Supporting information


**Appendix S1:** Supplementary Information

## Data Availability

Data can be made available upon reasonable request.
