# Supplementary material for: DNA methylation markers for cancer risk prediction of vulvar intraepithelial neoplasia
Source: Int J Cancer. 2021 Jan 19;148(10):2481–8. doi: 10.1002/ijc.33459 (PMC8048962; doi:10.1002/ijc.33459)
Supplement: Supplementary file 1 — Appendix S1: Supplementary Information [file IJC-148-2481-s001.pdf]

DNA methylation markers for cancer risk prediction of vulvar intraepithelial neoplasia

Nikki B. Thuijs[1], Johannes Berkhof[2], Müjde Özer[3], Sylvia Duin[1], Annina P. van Splunter[1], Barbara C. Snoek[1], Daniëlle A.M. Heideman[1], Marc van Beurden[4], Renske D.M. Steenbergen[1,*], Maaike C.G. Bleeker[1,*]

Table of contents

Supplementary Figure 1

Supplementary Figure 2

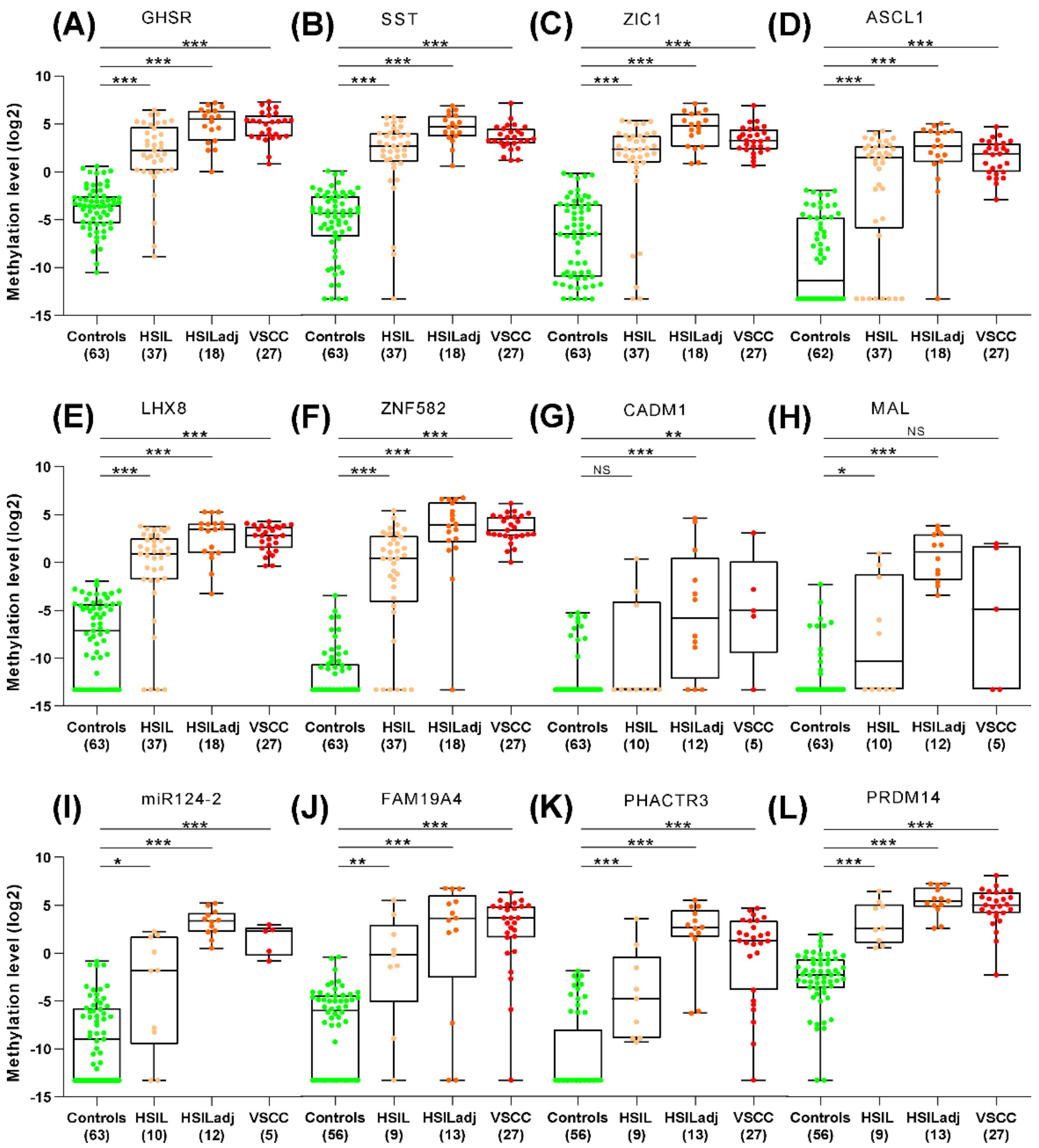

Supplementary Figure 1. DNA methylation levels shown relative to the reference gene *ACTB* (log2-transformed Δ(Δ)Ct ratios; y-axis) for HPV positive samples for the 4 disease categories (x-axis) for 12 markers: (A) GHSR, (B) SST, (C) ZIC1, (D) ASCL1, (E) LHX8, (F) ZNF582, (G) CADM1, (H) MAL, (I) miR124-2, (J) FAM19A4, (K) PHACTR3, and (L) PRDM14. Differences between histological categories upon Kruskal-Wallis test, followed by post hoc testing using the Mann-Whitney U test and Bonferroni multiple testing correction: *p<.05 (marginal evidence), **p<.01 (moderate evidence), ***p<.001 (strong evidence), NS: not significant. Abbreviations: HSIL = high-risk squamous intraepithelial lesion, HSILadj = HSIL adjacent to VSCC, VSCC = vulvar squamous cell carcinoma.

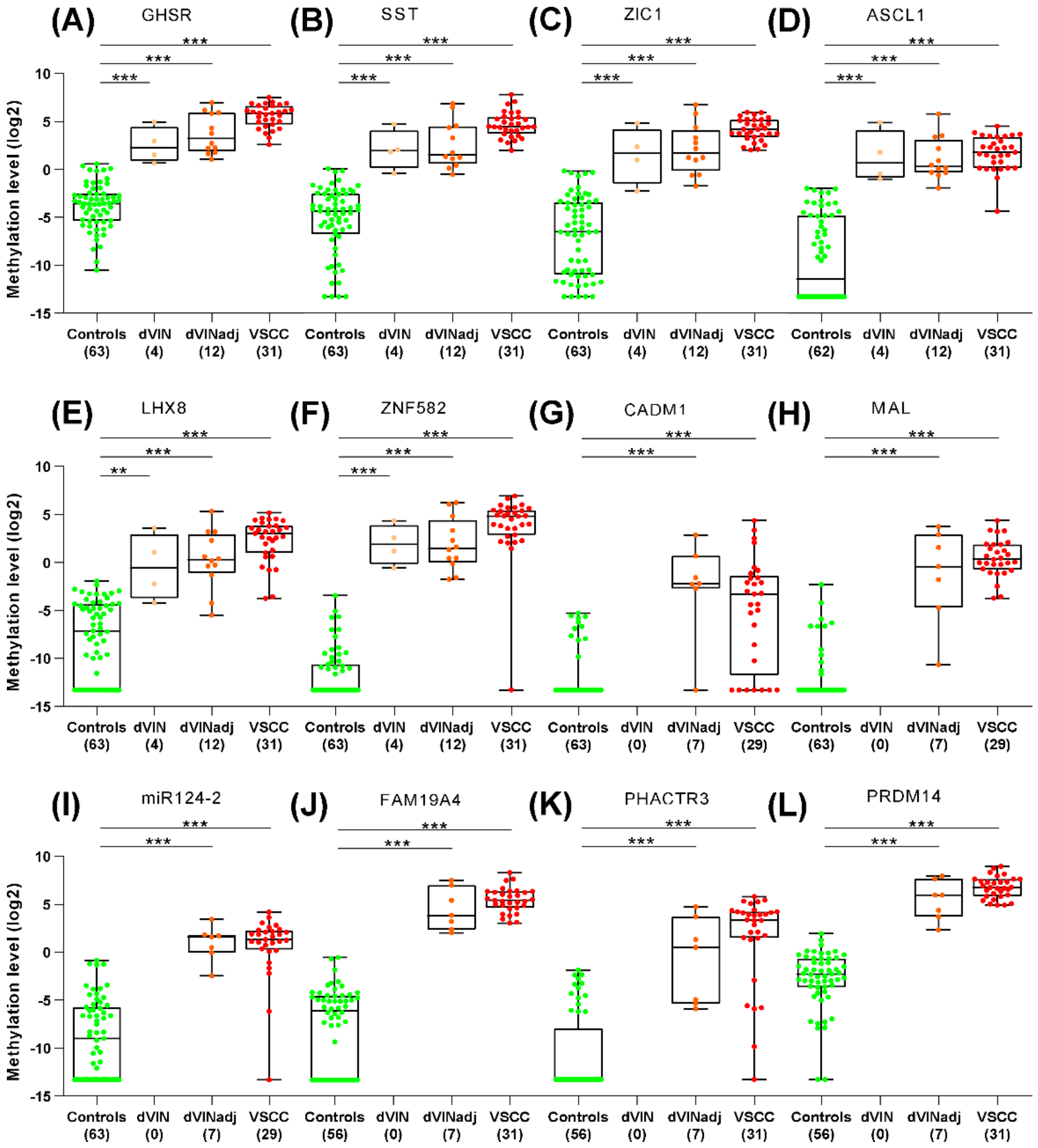

Supplementary Figure 2. DNA methylation levels shown relative to the reference gene *ACTB* (log2-transformed Δ(Δ)Ct ratios; y-axis) for HPV negative samples for the 4 disease categories (x-axis) for 12 markers: (A) GHSR, (B) SST, (C) ZIC1, (D) ASCL1, (E) LHX8, (F) ZNF582, (G) CADM1, (H) MAL, (I) miR124-2, (J) FAM19A4, (K) PHACTR3, and (L) PRDM14. Differences between histological categories upon Kruskal-Wallis test, followed by post hoc testing using the Mann-Whitney U test and Bonferroni multiple testing correction: *p<.05 (marginal evidence), **p<.01 (moderate evidence), ***p<.001 (strong evidence), NS: not significant. Abbreviations: dVIN = differentiated high-grade vulvar intraepithelial neoplasia, dVINadj = dVIN adjacent to VSCC, VSCC = vulvar squamous cell carcinoma.
